# Recent advances in the production of recombinant glycoconjugate vaccines

**DOI:** 10.1038/s41541-019-0110-z

**Published:** 2019-05-01

**Authors:** Emily Kay, Jon Cuccui, Brendan W. Wren

**Affiliations:** Department of Pathogen Molecular Biology, London School of Hygiene and Tropical Medicine, London, WC1E 7HT UK

**Keywords:** Immunology, Pathogenesis

## Abstract

Glycoconjugate vaccines against bacteria are one of the success stories of modern medicine and have led to a significant reduction in the global occurrence of bacterial meningitis and pneumonia. Glycoconjugate vaccines are produced by covalently linking a bacterial polysaccharide (usually capsule, or more recently O-antigen), to a carrier protein. Given the success of glycoconjugate vaccines, it is surprising that to date only vaccines against *Haemophilus influenzae* type b, *Neisseria meningitis* and *Streptococcus pneumoniae* have been fully licenced. This is set to change through the glycoengineering of recombinant vaccines in bacteria, such as *Escherichia coli*, that act as mini factories for the production of an inexhaustible and renewable supply of pure vaccine product. The recombinant process, termed Protein Glycan Coupling Technology (PGCT) or bioconjugation, offers a low-cost option for the production of pure glycoconjugate vaccines, with the in-built flexibility of adding different glycan/protein combinations for custom made vaccines. Numerous vaccine candidates have now been made using PGCT, which include those improving existing licenced vaccines (e.g., pneumococcal), entirely new vaccines for both Gram-positive and Gram-negative bacteria, and (because of the low production costs) veterinary pathogens. Given the continued threat of antimicrobial resistance and the potential peril of bioterrorist agents, the production of new glycoconjugate vaccines against old and new bacterial foes is particularly timely. In this review, we will outline the component parts of bacterial PGCT, including recent advances, the advantages and limitations of the technology, and future applications and perspectives.

## Introduction

A defining characteristic of a successful vaccine is the ability to evoke long-lasting protective immunity with minimal side effects. The most successful human vaccines are often glycoconjugates, which are combinations of a protein added to a sugar glycan. Coupling a glycan to a protein results in multiple triggers for the immune system; creating long-term immunological memory and increasing vaccine stability. Examples of current glycoconjugate vaccines^1^ fully licensed for human use include those for *Haemophilus influenzae* type B,^2,3^ *Neisseria meningitidis*^4^ and some *Streptococcus pneumoniae*^5^ strains. A major advantage of glycoconjugate vaccines over many existing vaccines is that they are suitable for most human populations, including infants and the elderly.^6^

**Fig. 1:**
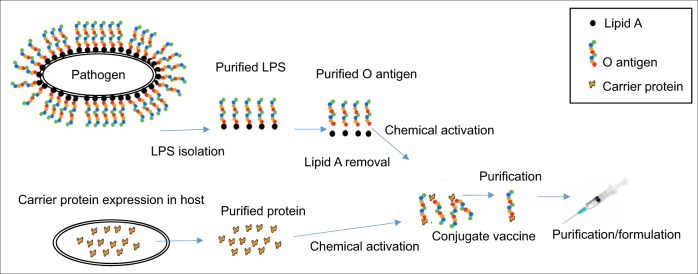
Traditional chemical conjugation method for the production of glycoconjugate vaccines. Multiple steps are required whereby the O-antigen must be purified from the pathogen of interest, detoxified and subject to chemical activation. In parallel, the protein must also be purified and chemically activated before protein and glycan can be conjugated. Following conjugation, further rounds of purification are necessary before vaccine can be administered

The global glycoconjugate vaccine market is projected to be worth approximately US $10 billion by 2020. Glycoconjugate vaccines are traditionally made by chemical activation of the polysaccharide at random sites, or at the reducing end,^7^ followed by conjugation to a carrier protein. This process requires initial steps to purify the glycan polysaccharide from the pathogenic organism, against which the vaccine is targeted, including removal of contaminating endotoxin, and purification of the acceptor protein from the organism of choice. For most licenced vaccines, the carrier protein is de-activated *Corynebacterium diphtheria* toxin 197 (CRM197), which has recently been made available as a cloned product.^8^ Overall, production via traditional chemical coupling of glycoconjugate vaccines is a multistep process and requires several rounds of purification to ensure that the glycoconjugate has been assembled correctly (Fig. 1). Although existing glycoconjugate vaccines are safe and effective, they have significant shortcomings; they are prone to batch-to-batch variation, have a reduction in efficacy over time due to glycan/serotype replacement, and are expensive to produce. Vaccine cost is a major concern for the Global Alliance Vaccine Initiative (GAVI) who have funded >143 million pneumococcal childhood immunisations in nearly 60 countries. A significant part of this cost for the production of the 13-valent pneumococcal vaccine is due to manufacture with around 700 quality control tests required prior to sale.^9^

In recent years, significant progress has been made in the chemical synthesis of glycoconjugate vaccines and the use of outer membrane vesicles (OMVs) or glycosylated OMVs (glycOMVs) as vaccines.^10–12^ In the case of glycOMVs, the intrinsic adjuvant properties of OMVs and the flexibility of lipid A choice holds promise for the generation of a self-adjuvanting, non-toxic delivery system for carbohydrate antigens.^13,14^ Discussions on these strategies is beyond the scope of this review, which will focus on the construction and production of recombinant glycoconjugate vaccines by glycoengineering using Protein Glycan Coupling Technology (PGCT). For reviews on chemical approaches to production of glycoconjugate vaccines we refer the reader to the following articles.^15–17^

## The evolution of bacterial Protein Glycan Coupling Technology

The original genome sequencing of the human gastrointestinal pathogen *Campylobacter jejuni* (strain NCTC 11168) in early 2000 confirmed that a genetic locus hypothesised to be involved in general protein glycosylation^18^ was independent of the lipooligosaccharide and flagellar O-linked glycosylation loci.^19^ Central to this region was *pglB*, which encodes the oligosaccharyltransferase (OST), termed CjPglB, which was found to have significant sequence similarity to the eukaryotic Stt3p protein, an essential component of the eukaryotic OST complex.^20,21^

Functional analyses of the genes within the glycosylation locus revealed the structure of the substrate glycan and that it is assembled on the lipid carrier undecaprenyl pyrophosphate (Und-PP), which is present in the inner membrane.^22^ Und-PP naturally functions as an anchor for the scaffold construction of oligosaccharides and polysaccharides, including O-antigens in Gram-negative bacteria. The next significant step was the demonstration that CjPglB was also able to transfer a range of polysaccharides, such as structurally different bacterial O-antigens, to *C. jejuni* carrier proteins such as AcrA.^23^ At about the same time, an extended glycosylation sequon, D/EXNYS/T, recognised by CjPglB was identified. This sequon could be engineered into a flexible secondary structure within a given carrier protein^24^ or multiple sequons could be engineered at either the C- or the N-terminus of carrier proteins (“glycotags”).^25^

These fundamental studies into the mechanism and properties of the *C. jejuni N*-glycosylation system laid the groundwork for development of the first bacterial glycoengineering system. Key features of the system are, the ability to transfer a relatively broad range of sugar substrates, and the targeting of heterologous proteins for site-specific glycosylation using a short sequence motif. Collectively, these studies provided the requisite ingredients for making customised recombinant glycoconjugate vaccines by PGCT. However, a drawback is the requirement for the presence of an acetamido group modification on the reducing end sugar in order for the glycan to be transferred by CjPlgB.

## The component parts for the production of recombinant glycoconjugate vaccines

**Fig. 2:**
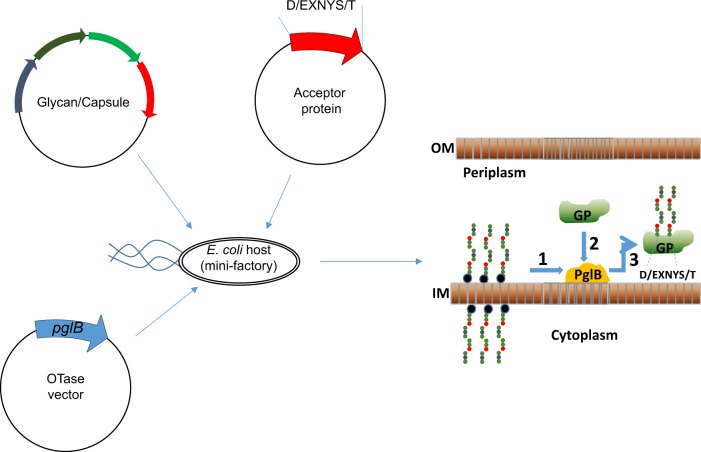
Glycoengineering approach to the production of glycoconjugate vaccines. An *E. coli* cell is transformed with three plasmids to generate the glycoconjugate protein (GP). PGCT occurs in three stages: stage 1; Glycan expression, stage 2; Carrier protein design and expression, stage 3; Coupling. The polysaccharide is synthesised on an undecaprenol pyrophosphate lipid anchor (blue/black circle) within the cytoplasm; this is transferred to the periplasmic compartment where PglB recognises the lipid linked reducing end sugar and transfers the polysaccharide en bloc onto an acceptor-sequon (D/E-X-N-X-S/T) on the carrier protein to produce the GP. IM, inner membrane; OM, outer membrane. This figure is adapted from Cuccui et al.^70^

PGCT can be divided into three stages summarised in Fig. 2:

### Glycan expression

**Fig. 3:**
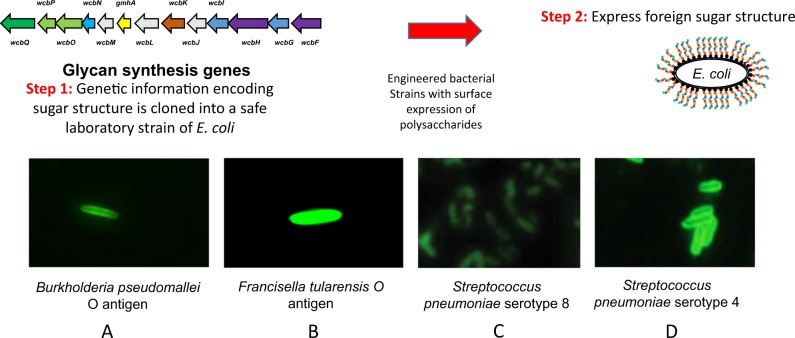
Glycan expression technology (GET). To express foreign sugar structures in *E. coli*, first the genes responsible for synthesising the glycan must be cloned. The example here is CPS I coding locus of *B. pseudomallei* K96243. Expression of these genes leads to synthesis and export of the foreign sugar attached to lipid A, in the absence of the native O-antigen from *E. coli*. **a**–**d** show Immunofluorescence microscopy of *E. coli* cells coated with the new sugar structures. Cells were probed with group or type-specific anti-glycan antibodies and Alexa Fluor 488 conjugated secondary antibody. A, unpublished; B, reproduced from Cuccui et al.^70^; **c**, **d** reproduced from Kay et al.^66^

The first stage in consideration of the construction of a recombinant glycoconjugate vaccine is to be able to clone and express the glycan of interest, which is usually a general glycosylation locus (e.g., *C. jejuni pgl*), an O-antigen, or a capsular polysaccharide. This process has been termed Glycan Expression Technology (GET), and because of its general importance for the production of glycoconjugate and glycOMV vaccines, has become a specialised area of glycoengineering in its own right. In many instances, the cloning and expression of a glycan is straightforward, where a specific gene cluster has all the requisite genetic information that can be transferred to a suitable host cell for expression. The glycan locus is usually introduced on a low copy plasmid to the *E. coli* host where its expression can be verified on the cell surface using specific antisera or lectins (see examples in Fig. 3). The reduction in price of DNA synthesis, and the ability to synthesise large regions of DNA, offers a more rapid approach to the cloning of polysaccharides.

However, a potentially more convenient approach is to use the bacteria from which the glycan originated, and to introduce the carrier protein and coupling enzyme into the native host cell (e.g., *Salmonella paratyphi* A) for recombinant expression.^26,27^ Another alternative would be to add in pathways from other more closely related bacteria for optimum expression; such as using surrogate *Pseudomonas aeruginosa* genes to reconstitute a *Staphylococcus aureus* capsule in *E. coli*.^28^

### Carrier protein design and expression

The identification and characterisation of the extended glycosylation sequon (D/EXNYS/T) for CjPglB, along with the advent of DNA synthesis means glycosylation sites can be readily engineered into a target carrier protein. Insertion of the sequon within a flexible region of a target protein results in efficient glycosylation by PglB,^24,29^ although the efficiency is known to vary depending on location of the sequon.^30^ This opened up the possibility that almost any protein could be modified to become a carrier for the target glycan. This initially meant that a given carrier protein’s structure needed to be known, as inaccessible regions would lead to reduced glycosylation efficiency. However, studies that are more recent have indicated that the D/EXNYS/T sequon can be added to the termini of a carrier protein, and that multiple sequons can be added for the production of a more heavily glycosylated vaccine.^25,31^

To date it is remarkable that glycoconjugate vaccines have relied on just CRM197, tetanus toxoid, and more recently, *H. influenzae* protein D.^32,33^ It could be argued that there will be a need for different carriers if multiple glycoconjugate vaccines are licenced and given to an individual. Using the same carrier for different glycoconjugate vaccines may result in the immune response dampening the effect of subsequent vaccination.^34,35^ The ease of DNA synthesis and the introduction of specific glycosylation sites allows enormous variety in the choice of carrier protein. Questions can now be answered as to whether any given carrier protein has a “bystander effect” upon immunisation.

Recent PGCT-derived glycoconjugate vaccines contain both the glycan and protein from the same pathogen, a so called “double-hit” approach for vaccination, where the carrier protein could play a more active immunological role.^36^ This may be particularly useful for bacterial species with multiple, diverse glycan structures where it would be difficult to protect against all glycan variants (e.g., pneumococcal vaccines). In addition, if the protein carrier was from another pathogen and had immunological properties in its own right, then dual protection against two infections may be possible from a single recombinant glycoconjugate vaccine. However, the proven ability of CRM197 to act as a suitable carrier without interfering with the protective properties of the vaccine has stood the test of time, and licensing alternative carrier proteins will require significant evaluation.

### Coupling

Since the original identification of CjPglB numerous orthologues have been identified, largely among the epsilon-proteobacteria subdivision of bacteria. Indeed, some bacteria have two PglB paralogues.^37^ The quest for alternatives to CjPglB are to: (i) broaden the range of glycans that can be coupled, (ii) have more active enzymes with higher efficiency for coupling and (iii) have PglB enzymes that recognise alternative carrier protein sequons. For example, PglBs have been identified from two species of *Desulfovibrio* that did not require the negatively charged amino acid at position-2 and were, therefore, able to glycosylate a shorter N-X-S/T sequon.^38,39^ Similarly, using PglB orthologues from the deep-sea vent epsilon-proteobacteria demonstrated altered specificity in the OST enzymes.^40^

In 2011 the crystal structure of the PglB enzyme of *Campylobacter lari* was determined, which has acted as a guide for a directed evolution approach to modify the functional characterisation of CjPglB orthologues, with the goal of relaxing the acceptor-sequon specificity.^41^

## Extending the capabilities of PGCT

In the 18 years since its inception, glycoengineering in bacteria using GET and PGCT has come a long way. It was originally thought that CjPglB had an absolute requirement for the presence of an acetamido group modification on the reducing end sugar in order for the glycan to be transferred. However, an exception to this restriction was provided with the transfer of the *Burkholderia pseudomallei* K96243 O-antigen repeating unit with the structure -3-)-β-d-glucopyranose-(1–3)-α-L-6-deoxy-talopyranose-(1- to the *C. jejuni* carrier protein AcrA, where talose was the reducing end sugar.^42^ In order to achieve this transfer, a *wecA* and *waaL* deletion strain was constructed (*E. coli* SDB1). A likely explanation as to why this strain enabled transfer of a typically non-permissive sugar is that, in the absence of metabolic load competition from the enterobacterial common antigen and ligase pathways, the Und-PP lipid carrier shunted the *B. pseudomallei* O-antigen to PglB for glycosylation of the acceptor protein. Therefore, under favourable conditions, CjPglB can transfer glycans without an acetamido modification on the reducing end sugar.

In 2015, Ihssen et al.^43^ reported a structure guided directed evolution approach for CjPglB in order to enhance its ability to transfer glycans that lack *N*-acetyl sugars. This work resulted in the modification of the amino acids S80R, Q287P and N311V in CjPglB. When this modified O-Tase was introduced into *Salmonella enterica* LT2 for a typhoid vaccine conjugate, transfer of the LT2 O-antigen was evident, even though the reducing end sugar is galactose. More recently, the structure of PglB bound to a reactive LLO has been reported providing more information about the close interactions within the active site.^44^

A further limitation to CjPglB appears to be related to the structure of adjacent sugars to the end group of the polysaccharide being transferred. It has been reported that PglB is unable to transfer polysaccharides where the two sugars proximal to the lipid carrier are connected via a β-(1 → 4) linkage.^45^ However, in their study dolichol, a non-native isoprene carrier, was used instead of undecaprenol. It may be that CjPglB exhibits selectivity toward the isoprene carrier; dolichol contains more isoprene units and also lacks a double bond, compared to undecaprenol, which would afford dolichol saccharides greater rotational mobility.^46^ If linkage were the sole reason why some polysaccharides cannot be transferred, then there is a potentially significant technological restriction, as the majority of *S. pneumoniae* serotypes have this β-(1 → 4) linkage. However, there is evidence that the transfer limitation may be more complex than just linkage. Valderrama-Rincon et al. demonstrated utilisation of CjPglB to create humanised glycoproteins, where the two sugars nearest the lipid carrier are GlcNAc β1 → 4 GlcNAc.^47^ In their initial study glycosylation efficiency was low ( < 1% protein glycosylated) but subsequent efforts have increased efficiency of protein glycosylation tenfold.^48^

PGCT is not limited to using PglBs, and new enzymes are continually being discovered that extend the capabilities of biological conjugation further. In recent years, *O*-linked OTases, often referred to as PglL, have been discovered and their functions characterised, in a similar manner to the early PglB work. The PglL family of enzymes demonstrate increased substrate promiscuity relative to PglBs. They do not require an acetamido group modification at the reducing end of a polysaccharide. The greatest challenge with this system appears to be: (i) controlling this promiscuity by generating *E. coli* strains with minimal interfering polysaccharide biosynthetic pathways, and (ii) reducing the size of the amino acid acceptor-sequon. In elegant studies by the Wang group, they demonstrated transfer of the O-antigen of *Salmonella paratyphi* A (which has a galactose reducing end sugar) to a modified cholera toxin B subunit by the *Neisseria meningitides* O-Tase, PglL.^26,27^ Here, a fragment of *N. meningitides* PilE, (45SAVTEYYLNHGEWPGNNTSAGVATSSEIK73) in which Ser63 was the native glycosylation site,^49^ was fused to the C-terminus of the cholera toxin B subunit. Previous studies reported that the use of short polysaccharide chains may not induce a sufficient immune response.^50–53^ In a subsequent study, the group addressed this by deleting the gene responsible for controlling the length of the polysaccharide chain, *cld*. This resulted in a recombinant *S. paratyphi* A O-antigen glycoconjugate with a chain length of ~20 repeats that demonstrated excellent immunogenic properties.^27^ Recently, it has been demonstrated that a newly identified *O*-linked OST from *Acinetobacter* species, PglS, can couple *S. pneumoniae* capsular polysaccharides with glucose as a reducing end sugar, which could not be coupled with CjPglB.^54^ This paves the way for a polyvalent vaccine produced using bioconjugation as ~70% of serotypes have glucose at the reducing end^55^; including 9/13 serotypes in the currently licensed pneumococcal Prevnar13 glycoconjugate vaccine.

In addition to OSTases, a cytoplasmic glycosyltransferase in the GT41 family (NGT) from *Actinobacillus pleuropneumoniae*, has been demonstrated to create *N*-glycosidic linkages at asparagine residues, within an NXS/T sequon with UDP-glucose as the substrate.^56–58^ Much of the research with this enzyme has focused on biochemical characterisation and its potential for vaccine assembly is currently being explored.^59^

## Considerations of the host cell

The ability to engineer commonly used laboratory strains such as *E. coli* to efficiently synthesise non-native sugar structures by the recombinant expression of enzymes from different carbohydrate biosynthesis pathways is a pre-requisite for efficient product yield, scaling up and vaccine production. It is necessary for *E. coli* (or other bacterial hosts) to have the requisite biosynthetic machinery to generate such products. Therefore, depending on a range of factors including removing interfering pathways and streamline expression of the cloned glycan, the degree of polymerisation for O-antigen or capsular polysaccharides, and whether the native sugars are present, it may be necessary to use a range of specifically mutated and adapted *E. coli* strains.

It is often desirable to have some of the competing glycan production pathways from *E. coli* removed to reduce metabolic load. Such mutants with major glycan structures removed are available to the community and include the SCM strain series: SCM3^60^ and SCM6 (constructed by the Valvano lab), and SCM7 (constructed by the Aebi lab) from SΦ874.^61^ Also, CLM24^23^ and CLM37 strains,^22^ with targeted disruption of *waaL* and *wecA*, respectively are useful for glycoengineering. The SCM strains have large regions of the chromosome deleted, including the entire *rfb* and *wca* clusters, destroying O-antigen and colanic acid coding regions. However, although this may help in terms of metabolic streamlining, in some instances these regions may contain pathways that form the necessary precursors for the expression on the target glycan (e.g., some *S. pneumoniae* capsule serotypes, with rhamnose biosynthesic pathways^61^). A more targeted approach has been described recently, whereby the native polysaccharide gene clusters are replaced with pathways for recombinant glycan expression. This allows for similar glycosylation efficiency but higher glycoprotein yields due to enhanced growth dynamics.^62^

One example of improving PGCT has been the notion that overexpression of *pglB* can be harmful to the host cell. PglB is a 13-transmembrane enzyme and it has been reported that overexpression actually reduces total protein yield.^63^ This result confirms that biological conjugation is a process that requires a balance between optimal glycosylation and overall protein yield. This balance is likely to require fine-tuning when glycan and protein combinations are changed, as polysaccharides can place different metabolic burdens on the host *E. coli* cell. We have recently demonstrated how *S. pneumoniae* serotype 4 capsular polysaccharide production within *E. coli* can be significantly enhanced by removing the function of the native *wecA* gene (encoding the UDP-*N*-acetylglucosamine Und-PP transferase) and replacing it with the UDP-glucose-4-epimerase encoding gene, *gnE* from *C. jejuni* (unpublished data).

Another example of tailoring the *E. coli* strain for glycoconjugate production and purification is to reduce toxicity from co-purified host elements such as lipid A. Removal of the myristoyl group from lipid A (*lpxM* mutation) readily reduces endotoxicity of the vaccine product with retention of protective efficacy in mice.^64,65^

## Current developments of recombinant glycoconjugate vaccines

Glycoconjugate vaccines and vaccine candidates produced using PGCT fall into three general areas of development:

### Improving existing vaccines

*Streptococcus pneumoniae* is a globally important cause of infectious disease, responsible for a high proportion of cases of pneumonia, meningitis and sepsis. Although existing vaccines based on capsular polysaccharide antigen chemically conjugated to a protein carrier are effective, they have significant shortcomings, including expense, limited serotype coverage, lack of flexibility in altering target serotypes, and reduction in efficacy over time due to serotype replacement. It is estimated that the global pneumococcal vaccine market, which includes 10 and 13-valent glycoconjugate vaccines, is £5 billion, but still a reported estimate of nearly one million infants die each year of pneumococcal disease where at least an equivalent is burdened with the disease through long-term complications. The commercial 10- and 13-valent vaccines largely use CRM197 as the chemically conjugated carrier protein. To date several capsules have been expressed in *E. coli* using GET^66^ and capsule serotype 4 has been coupled to the *C. jejuni* AcrA carrier protein by PGCT.^67^ This vaccine has been shown to protect against homologous challenge in the murine pneumococcal infection model.^67^ It is possible that PGCT could be exploited to produce vaccines equivalent to those that are currently licenced at lower cost, or with increased valency. However, there are limitations, including which capsular polysaccharides can be efficiently expressed in *E. coli* and the fact that most *S. pneumoniae* capsular serotypes are incompatible with the coupling enzyme CjPglB.

An alternative approach being developed is to use conserved *S. pneumoniae* proteins as carriers for capsular polysaccharide antigens, which may provide heterologous protection against non-vaccine capsular serotypes and improve mucosal defences by stimulating Th17 immunity. Using this double-hit approach it has been shown that PGCT can make *S. pneumoniae* protein/capsular antigen glycoconjugates that, in murine models, induce similar levels of anti-capsular antibody to existing vaccines, as well as strong anti-protein antigen responses that recognise heterologous capsular serotypes.^36^ Additionally, the expression of multiple capsular polysaccharides serotypes in *E. coli* opens up the possibility of glyOMVs as a vaccine alternative. With these recent results, it is likely that GET and PGCT will collectively deliver a broad-coverage, effective and low-cost *S. pneumoniae* glycoconjugate vaccine with novel protein/capsule combinations.

The ability to ‘graft’’ the sequon onto various parts of a protein means that PGCT is suitable to create a new generation of knowledge-based glycoconjugate vaccines, to maximise activation of the adaptive immune response. A model, proposed by Avci et al.,^68^ suggests that a glycoconjugate vaccine is internalised by antigen presenting cells, and that once inside, the vaccine undergoes degradation into shorter glycan polymers, but also that the protein would be broken down into peptides. These short glycopeptides are capable of binding directly to MHC class II receptors and were presented/recognised by a specific subpopulation of T cells (known as carbohydrate specific T cells or Tcarbs). More recently, this Tcarb dependent concept was demonstrated for other glycoconjugates including the Vi antigen of *Salmonella Typhi*, the CPS of type Ib group B streptococci, and the CPS of *H. influenzae* type b.^69^ PGCT is ideally suited to the glycosylation of specific sites on an acceptor peptide, as a parallel to the four-amino acid addition used in the GBSIII-OVAp study, the PglB targeting sequence D/EXNYS/T can be grafted onto an acceptor peptide. Thus, the potential of the technology is to reduce vaccination costs, not only through the biological conjugation process, but by also creating more potent antigens, with the possibility of reducing vaccine dosage. We, and others have also recently demonstrated that, depending on the polysaccharide, polymerisation can be controlled when the glycan is being heterologously expressed in the *E. coli* cell. Thus, in several cases we can assemble a range of polymer lengths from short up to lengths exhibited by the native organism. Interestingly, not all glycoconjugates are Tcarb dependent and, it was recently shown that, the group C polysaccharide of the *N. meningitidis* vaccine appears to require a different mechanism of presentation.^69^

### New vaccines

For many bacterial diseases, including high-threat pathogens, there are no current protective vaccines available. To address this unmet medical need, there has been a recent surge in the construction of tailor made glycoconjugate vaccines against a range of pathogens using PGCT. Examples of high-threat pathogens include *Francisella tularensis*, where a single O-antigen was coupled to *Pseudomonas* ExoA carrier protein and was shown to protect in the murine infection model.^70^ More recently the ExoA construct was modified with seven O-antigen repeats using glycotags. This second generation, more heavily glycosylated vaccine, was shown to be considerably more protective in both the murine and rat infection models of tularaemia.^31^ Similarly, O-antigen conjugate constructs have been shown to protect against the high-threat pathogen *Burkholderia pseudomallei*, in the murine meliodosis infection model.^42^ Elegant glycoengineering studies have been used to construct a *Staphylococcus aureus* capsular conjugate with ExoA that protects against infection in a murine model.^28^ Some vaccines produced by PGCT have entered clinical trials, including O-antigen conjugates for *Shigella dysenteriae* type I,^71^ *Shigella flexneri* 2a,^72^ and uropathogenic pathogenic *E. coli*^73^ (Table 1). A wider selection of possible glycoconjugate vaccines has recently been reviewed.^1^

**Table 1:** Current glycoconjugate vaccines is developed using PGCT

Organism	Glycan	Protein carrier	Status	Manufacturer	References
*Streptococcus pneumoniae*	Capsule-multivalent	rEPA	Phase I clinical trials	Limmatech Biologics	NCT03303976^a^
*Streptococcus pneumoniae*	Capsule-serotype 4	piuA	Development	Academic- UCL/LSHTM UK	Reglinski et al.^36^
*Staphylococcus aureus*	Capsule-Type 5 and 8	rEPA	Development	GlycoVaxyn	Wacker et al.^28^
*Shigella dysenteriae*	Capsule-Type 1	rEPA	Phase I clinical trials	Limmatech Biologics	Hatz et al.^71^
*Shigella flexneri*	Capsule- 2a	rEPA	Phase I clinical trials	Limmatech Biologics	Riddle et al.^72^
*Escherichia coli*	O-antigen-ExPEC serotypes 01, 02, 06, 025	rEPA	Phase Ib clinical trials	Limmatech Biologics/ J&J	Huttner et al.^79^
*Francisella tularensis*	O-antigen	rEPA	Development	Government/ Academic -DSTL/ LSHTM UK	Marshall et al.^31^
*Burkholderia pseudomallei*	O-PSII	AcrA	Development	Government/ Academic- DRDC/ University of Alberta Canada	Garcia-Quintanilla et al.^42^

PGCT-adapted from Micoli^1^

^a^ClinicalTrials.gov Identifier

### New opportunities

Given the foreseen reduction in the costs of recombinant glycoconjugate vaccines made using GET and PGCT, it is possible to consider glycoconjugate vaccines for the livestock and animal markets, where low cost is the major driving force in vaccine development. Apart from protecting animals against disease and the associated economic advantages, vaccines against zoonotic pathogens will directly benefit human health and reduce the use of antibiotics in the livestock industry. Some concepts currently being explored in our laboratory include, creating live attenuated organisms that deliver a payload of a glycoconjugate vaccine. This ‘trojan horse’’ approach is particularly attractive as it promises to match current manufacturing costs and storage conditions, whilst providing protection against additional pathogens.

Biological conjugation is also being explored in the creation of humanised glycoproteins. This process has been described as a ‘bottom up approach’’, as bacterial cells have yet to be discovered that create the human pentasaccharide core glycan. For this research, PglB variants have been created and tested using a process termed GlycoSNAP, with the aim of being able to transfer the human pentasaccharide core glycans to NXS/T.^74^ This also increases the range of potential carrier proteins that can be used with PGCT. Similarly, GlycoSCORES is a systematic platform for glycosylation sequence characterisation and optimisation, by rapid expression and screening, that has been used to identify glycosylation consensus sequences in other glycosyltransferases, including the cytoplasmic *N*-linked glycosylation system NGT.^75^

## Conclusion and future perspectives

Infections caused by bacterial pathogens, especially antibiotic-resistant ones, are an ever-increasing public health threat and global problem. Glycoconjugate vaccines are among the most effective means in combating such infections. To date, glycoconjugate vaccines have had a huge impact on global infant mortality and morbidity. Glycoconjugate vaccine production using PGCT has enormous potential to make a significant impact on bacterial disease, both in humans and animals. The proof of principle experiments have been completed, and a number of vaccines are now in clinical trials. The evidence is mounting that the technology works, and further technical modifications are being developed to improve yield, the quality of the products and the flexibility in design.

It is perhaps surprising that we still don’t fully understand why glycoconjugates are such effective vaccines, particularly in infants. The Kasper group have presented a plausible model where the glycoconjugate bound to a T-cell receptor is internalised and, in the endosome, is broken down into smaller peptides.^68^ Both the peptide and polysaccharide are trimmed, allowing for binding of a glycopeptide to major histocompatibility complex class II and presentation of this moiety to the T cell. This model appears to answer the long-standing question as to why co-administered protein and sugar, which are not covalently bound, fail to induce an optimal immune response.

Although covalent linkage is the basis of current licenced vaccines, there are immunological models, and prototype vaccines, being generated that are starting to indicate that there may be alternative ways in which T cell help may be elicited. A protein capsular matrix vaccine, where capsule is trapped in a protein matrix, produced an equivalent immune response in mice to a covalently linked conjugate vaccine,^76^ and alternative approaches such as GMMAs^77^ and OMVs,^10–12^ where the glycan is not covalently attached to protein, also appear to be successful in preparing the immune system. Conjugating polysaccharide to a lipid carrier has also been shown to provide protection in mouse studies.^78^ Further studies will be required to demonstrate how widely applicable these new approaches may be.

Given the plethora of diverse glycoconjugate vaccine candidates now available through PGCT, these models can now be put to the test. This may provide useful clues as to how current glycoconjugate vaccines could be improved, by rationally designing the protein carrier and the glycan attached to it. The more candidates that are evaluated, the greater contribution to a true understanding of how glycoconjugate vaccines work, and what makes a useful fully effective protein carrier. The resulting vaccine candidates will shed light on how the immune system responds to carbohydrates and provide mechanistic insight that can help guide future vaccine design. The full potential of glycoconjugate vaccines has not yet been reached.^6^ New vaccines on the horizon using PGCT include those against Groups A and B Streptococci, Brucella, EPEC, EHEC and traveller’s diarrhoea.^1^

Further developments in the glycoengineering toolbox will allow this exciting field to continue at the forefront of scientific discovery. Bacterial glycoengineering has now come of age; the glycotoolbox will continue to evolve with further opportunities for vaccinology and beyond. As carbohydrate-based vaccine formulations continue to be improved, so too will their clinical potential beyond pathogenic bacteria, into vaccines against viruses, parasites and cancer.
